# Interactions of organic acids with *Campylobacter coli* from swine

**DOI:** 10.1371/journal.pone.0202100

**Published:** 2018-08-10

**Authors:** Ross C. Beier, Roger B. Harvey, Charles A. Hernandez, Michael E. Hume, Kathleen Andrews, Robert E. Droleskey, Maureen K. Davidson, Sonia Bodeis-Jones, Shenia Young, Sara E. Duke, Robin C. Anderson, Tawni L. Crippen, Toni L. Poole, David J. Nisbet

**Affiliations:** 1 United States Department of Agriculture, Agricultural Research Service, Southern Plains Agricultural Research Center, Food and Feed Safety Research Unit, College Station, Texas, United States of America; 2 United States Food and Drug Administration, Office of Research, Center for Veterinary Medicine, Laurel, Maryland, United States of America; University of Campinas, BRAZIL

## Abstract

*Campylobacter coli* is a bacterial species that is a major cause of diarrheal disease worldwide, and *Campylobacter* spp. are among the top 5 foodborne pathogens in the United States. During food production organic acids (OAs) are often used to remove bacteria from animal carcasses. The interactions of six OAs with 111 *C*. *coli* strains obtained from swine and retail pork chops were studied by determining the molar minimum inhibitory concentrations (MIC_M_s) of the *C*. *coli* strains, and the pH at the MIC_M_s. The Henderson-Hasselbalch equation was used to calculate the concentrations of the undissociated and dissociated OAs at the MIC_M_s of the *C*. *coli* strains. The results for the 111 different *C*. *coli* strains obtained from different locations were treated as a single group for each OA since many of the *C*. *coli* strains behaved similarly to each different OA. Inhibition of *C*. *coli* was not dependent on pH or on the undissociated OA species, but *C*. *coli* inhibition correlated with the dissociated OA species. Therefore, if the concentration of the dissociated OAs decreases from optimum, one may then expect that *C*. *coli* bacteria would escape disinfection. The concentration of the dissociated OA should be carefully controlled in a carcass wash. We suggest maintaining a concentration of the dissociated acetic, butyric, citric, formic, lactic and propionic acids at 29, 23, 11, 35, 22 and 25 mM, respectively, when using a carcass wash with these OAs to remove *C*. *coli* bacteria. However, due to *C*. *coli* utilization of acetate, formate, lactate and propionate, these four OAs may not be the best choice to use for a carcass wash to remove *C*. *coli* contamination. Of the six OAs, citric acid was the most efficient at inhibiting *C*. *coli*.

## Introduction

*Campylobacter* spp. are Gram-negative, non-spore forming bacterial rods [1,2] that are a major cause of diarrheal disease in the United States [3] and throughout the world [1,4–8]. The Centers for Disease Control and Prevention (CDC) has estimated that each year there are 9.4 million domestically acquired foodborne illnesses, 55,961 hospitalizations, and 1,351 deaths due to 31 major pathogens in the United States [3,9]. *Campylobacter* spp. are among the top 5 foodborne pathogens in the United States, and they are estimated to be responsible for 845,024 illnesses, 8,463 hospitalizations, and 76 deaths each year [3,9]. *Campylobacter jejuni* and *C*. *coli* are the two main species most often associated with human foodborne illness in this genus [10–13], and they have a high % of DNA homology [14] and possess identical or highly related antigens [15]. In 2016, the CDC reported that *Campylobacter* and *Salmonella* caused the most reported bacterial foodborne illnesses in the United States [16]. In England during 2002 *C*. *jejuni* accounted for 93% of the reported cases and *C*. *coli* accounted for 7% of the reported cases [4]. Campylobacteriosis was the most often reported zoonosis in the European Union (E.U.) in 2015 [7]. The type and number of organisms in the E.U. illnesses during 2015 caused by *Campylobacter* spp. were primarily divided between *C*. *jejuni* and *C*. *coli* at 81.0% and 8.4%, respectively [7], although in France *C*. *coli* had a higher percentage of cases at 15.25% [13]. Therefore, *C*. *jejuni*, which is commonly found in poultry and poultry products, causes the most campylobacteriosis, and low levels of *C*. *jejuni* are also found in swine [17]. However, in some areas of the world the percentage of campylobacteriosis caused by *C*. *coli* may be as high as 35–40% [18]. *Campylobacter coli* is the predominant *Campylobacter* species found in the intestines of pigs and on pork products [19,20]. The impact of *C*. *coli* on infectious intestinal disease in humans has largely been ignored, even though *C*. *coli* is the second most common cause of human campylobacteriosis [21]. Most likely *C*. *coli* have been neglected as a human pathogen because of the predominance of *C*. *jejuni* campylobacteriosis [21]. Trace back investigations of *C*. *coli* foodborne outbreaks in Belgium (1995) [22], in Poland (2006) [23] and in Alaska (2013) [24] have all resulted in not determining the source of contamination. Epidemiologic and microbiologic data compiled by the Great Britain Public Health Laboratory Service (PHLS) Communicable Disease Surveillance Centre determined that risk factors for transmission of *C*. *coli* to humans are different compared to those for *C*. *jejuni* [4]. Therefore, this data shows a need to carry out species-specific studies, and develop separate strategies for control of these different organisms [21].

Comprehensive strategies to control foodborne pathogens throughout the food chain from the farm to the table are important [25]. A critical step in processing animals into food products is to wash the animal carcasses with organic acids (OAs) to remove surface bacteria. The OAs often used are acetic [26–28], citric [26], formic [27], lactic [26–31] and propionic acids [27,28]. Bacteria that are not removed from the carcass during the acid wash may later be found on the processed meat. Therefore, the efficacy of the acid wash step should be carefully evaluated.

It is believed that bacterial inhibition by OAs is dependent on pH or the undissociated acid species [32–35]; however, the specific mechanisms by which pH and OAs inhibit bacteria are not understood [36]. In our previous studies, molar values have been used for minimum inhibitory concentrations (MIC_M_s) when comparing pH, undissociated or dissociated acid forms because it allows an equivalent comparison of MIC results for acids with different molecular weights [37]. Previous studies evaluated *Escherichia coli* O157:H7 [37], *Pseudomonas aeruginosa* [38], non-O157 Shiga toxin-producing *E*. *coli* (non-O157 STECs) [39] and *Salmonella* enterica serovars [40] against OAs and clearly show that pH and levels of undissociated acids do not correlate with the MIC_M_s. However, levels of dissociated acids do closely correlate with the MIC_M_s. Also, a fully dissociable acid has been shown to cause the disintegration of the bacterial LPS layer [41]. During our previous studies it was observed that a decrease in the concentration of the dissociated acids may result in a large number of bacteria escaping disinfection [37–40].

In this present study, we describe the interactions of six different OAs with 111 *C*. *coli* strains, which were obtained in earlier studies that evaluated the pathogens in market age pigs [42], and food animals and retail meat [43]. Susceptibility studies of 111 *C*. *coli* strains to the OAs, acetic, butyric, citric, formic, lactic and propionic acids were conducted here. Comparisons are shown of the pH, undissociated acid species and dissociated acid species at the MIC_M_s of the *C*. *coli* strains.

## Materials and methods

### Ethics statement

No animals were utilized in this study. All *C*. *coli* strains were obtained from frozen stocks in glycerol as prepared by researchers in previous studies.

### *Campylobacter coli* and media

Previously, *C*. *coli* was isolated from cecal contents (n = 7), rectal swabs (n = 51) and feces (n = 5) of market age pigs [42], and *C*. *coli* also was previously isolated from cecal contents (n = 16) of market age pigs, from cecal contents of sows (n = 20) and from retail pork chops (n = 12) [43]. The above 111 *C*. *coli* strains were grown in our laboratory for 48 hours at 42°C on trypticase soy agar w/5% sheep blood BBL Stacker Plates (Becton, Dickinson and Company, Sparks, MD, USA) in a microaerobic atmosphere of 10% CO_2_, 5% O_2_, and 85% N_2_. For cryopreservation, the 111 *C*. *coli* strains were transferred from the BBL Stacker Plates and placed in FBP medium [44]. Briefly, FBP medium was made with Nutrient Broth (234000, Difco, Franklin Lakes, NJ, USA), Bacto™ Agar (214010, BD, Franklin Lakes, NJ, USA) at a final concentration of 0.12% (w/v), glycerol (49769, Fluka, Sigma-Aldrich, St. Louis, MO, USA) at a final concentration of 15% (v/v), and Bacto™ Yeast Extract (212750, BC, Franklin Lakes, NJ, USA) at a final concentration of 0.1% (w/v). The prepared FBP mixture was then autoclaved for 15 min at 121°C and 15 PSI and allowed to cool to 50°C in a water bath. Per label directions, *Campylobacter* Growth Supplement (SR0232E, Oxoid, Basingstoke, United Kingdom) was added to the cooled mixture. The prepared medium (1 ml) was added to each sterile cryogenic vial (5000–0020, Thermo Fisher Scientific, Houston, TX, USA). *Campylobacter* cells were added to the FBP medium at a turbidity of McFarland 3 to 4. The cells were then placed in a –80°C freezer for long term storage.

### Organic acid susceptibility testing

The OA MICs against the *C*. *coli* strains were determined by broth microdilution testing of fastidious bacteria according to the Clinical and Laboratory Standards Institute (CLSI) [45], and the methods presented by TREK Diagnostic Systems for susceptibility using *Campylobacter* sensititre plates [46]. Briefly, The *C*. *coli* strains were grown for 48 hours at 42°C, as described earlier. All *Campylobacter* susceptibility studies required incubation for 48 hours at 42°C either on trypticase soy agar w/5% sheep blood or in 96-well plates (U-bottom microplate, Greiner bio-one North America Inc., Monroe, North Carolina, USA) for broth microdilution testing because there were some strains that did not grow a sufficient amount in 24 hours to run the test. Several *C*. *coli* colonies were selected from the trypticase soy agar plates and diluted in 5 ml of Sensititre™ cation adjusted Mueller-Hinton broth w/TES (Remel Lenexa, KS, USA) to a 0.5 McFarland standard in a Nephelometer (TREK Diagnostic Systems Ltd., East Grinsted, UK). Since our experiments have a final total liquid volume of 100 μl in each well, to maintain a consistent bacterial concentration as suggested by the TREK Diagnostic Systems sensititre susceptibility test for *Campylobacter*, 200 μl of the 0.5 McFarland suspension was placed in tubes containing 11 ml of Sensititre™ cation adjusted Mueller-Hinton broth w/TES w/Lysed horse blood to provide 1 × 10^6^ CFU/ml. Following the proper dilution of OAs to 50 μl in each well of the 96-well plates [40], 50 μl of the lysed horse blood diluted bacteria was layered in all 96-wells of the microplate. Briefly, the OA dilutions consisted of 50 μl of each OA solution placed in wells 1 and 2, and the well 2 solution was diluted 1:2 across a 96-well U-bottom Greiner bio-one microplate through column 11, and column 12 was used as the positive control [40]. The bacteria filled microplates were covered with a perforated plastic adhesive cover sheet (YG522EA, Remel, Lenexa, KS, USA) and placed in a BD GasPak™ EZ standard or small incubation container (BD #260671 or BD #260002, respectively, Becton, Dickinson and Company, Sparks, MD, USA). BD GasPak™ EZ Campy Container System Sachets (BD #260680, Becton, Dickinson and Company, Sparks, MD, USA) were placed inside the incubation containers and the sealed containers were allowed to incubate for 48 hours at 42°C. MICs were determined as the lowest concentration of a compound that showed no visible growth of the organism [47] on a SensiTouch imaging system (TREK Diagnostic Systems Ltd., East Grinsted, UK). *Campylobacter jejuni* ATCC 33560 was used as a control organism for the OA susceptibility testing in the microaerobic atmosphere. These results were compared with results obtained from testing *Escherichia coli* ATCC 25922 in aerobic conditions, as ATCC 25922 was previously used as the control organism during aerobic OA testing [37–40].

Acetic acid was obtained from EM Science (Gibbstown, NY, USA). Butyric, citric, formic and propionic acids were obtained from Sigma-Aldrich (Milwaukee, WI, USA). Lactic acid was obtained from Alfa Aesar (Wad Hill, MA, USA). To make working solutions, the OAs were diluted with reverse osmosis water and then filter-sterilized using a 0.2 μm × 25 mm syringe filter (No. 431224, Corning Inc., Corning, NY, USA). The following concentrations of OAs were tested: acetic acid, 32–32,768 μg/ml; butyric acid, 16–16,384 μg/ml; citric acid, 16–16,384 μg/ml; formic acid, 16–16,384 μg/ml; lactic acid, 8–8,192 μg/ml; and propionic acid, 32–32,768 μg/ml.

### Determination of solution pH in 96-well plates at the *C*. *coli* MICs

Determination of pH was conducted as previously described [40]. Briefly, the pH was determined in three separate samples at each MIC for each OA, and then the means and standard deviations were determined. The solutions from 16-wells (1,600 μl) at the same MIC value for each OA were combined in a sterile 5 ml microtube (Argos Technologies, Inc., Vernon Hills, IL, USA). An Orion 3 STAR benchtop pH meter was used to measure the pH with a ROSS Ultra, glass combination pH electrode (Thermo Fisher Scientific, Chelmsford, MA, USA). Each pH determination at each MIC was conducted in triplicate.

### Calculation of the ratio of undissociated to dissociated acids

The Henderson-Hasselbalch equation can be used to calculate the concentration of conjugate base and undissociated weak acid [48]:

pH=pKa+log([A–][HA]) (1)

Where the pK_a_ is–log_10_ of the acid dissociation constant (K_a_), [A^–^] is the molar concentration of the conjugate base (or dissociated weak acid), and [HA] is the molar concentration of the undissociated weak acid [48]. The Henderson-Hasselbalch equation can be rearranged to provide the ratio of undissociated to dissociated acid [33]:

ratio=[HA][A–]=110pH–pKa (2)

Therefore, when the pK_a_ of a particular acid and the pH of the solution are known, then the ratio of the undissociated to dissociated acid can be calculated. The pK_a_ for acetic, butyric, citric, formic, lactic and propionic acid is 4.75, 4.82, 3.14, 3.75, 3.86 and 4.87, respectively. If the molar concentration of the acid is known, then the concentrations of the undissociated and dissociated acid species can be calculated from the ratio [37–40].

### Statistics

A contingency table association analysis was conducted on the data in Table 1 between the MIC_M_ values and sources. A Fishers Exact test (due to the small sample size) was used to assess for patterns requiring greater OA concentrations for control of *C*. *coli* strains from different sources.

**Table 1:** Organic acid MICs and MIC_M_s^a^ for 111 *Campylobacter coli* strains isolated from cecal contents, feces and rectal swabs of market age pigs, cecal contents of sows and from retail pork chops.

MIC (μg/mL)	MIC_M_ (mM)	Number of Bacteria from Swine				
		Market Age Pigs				
		Cecal	Feces	Rectal Swabs	Cecal (sows)	Pork Chops
**Acetic Acid**						
4096	68.2	–^b^	–	–	–	1
2048	34.1	19	4	40	14	5
1024	17.05	4	1	11	6	6
**Butyric Acid**						
2048	23.24	22	5	48	15	10
1024	11.62	1	–	3	5	2
**Citric Acid**						
2048	10.66	14	2	27	14	10
1024	5.33	9	3	24	6	2
**Formic Acid**						
2048	44.5	–	4	24	3	–
1024	22.25	23	1	26	17	12
512	11.12	–	–	1	–	–
**Lactic Acid**						
4096	45.47	1	–	1	3	4
2048	22.74	4	3	17	8	5
1024	11.37	18	2	32	9	3
512	5.68	–	–	1	–	–
**Propionic Acid**						
2048	27.65	16	5	36	13	8
1024	13.82	7	–	13	7	4
512	6.91	–	–	1	–	–
256	3.45	–	–	1	–	–

^a^MIC_M_s = Molar MICs.

^b^’–‘ = No observed MIC at this acid concentration.

## Results

The MICs and MIC_M_s obtained for *C*. *coli* strains against the OAs tested here are shown in Table 1. The *C*. *coli* MIC_M_s for acetic, butyric, citric, formic, lactic and propionic acids are similar for each individual acid whether the bacterial strains were obtained from market age pigs, sows or pork chops. *Campylobacter coli* strains from feces and rectal swabs of market age pigs required differential levels of OAs for control. The highest level of formic acid (44.5 mM) was required for inhibition of 50% of the feces and rectal swab strains. But a citric acid level of only 10.66 mM inhibited these same *C*. *coli* strains, which also was a lower acid concentration than the other OAs, acetic, butyric, formic, lactic, and propionic acids, except for lactic and propionic acids which inhibited 1 and 2 strains at levels of 5.68 and 6.91 mM, respectively. The highest level of an OA required for control of *C*. *coli* strains was for retail pork chop samples, which required 45.47 mM of lactic acid, and one strain required 68.2 mM acetic acid for inhibition. The lowest OA levels required for control of all strains was for citric acid (10.66 mM).

### Interplay of the six organic acids with respect to differential association for inhibition of *Campylobacter coli* from different isolation sources

Using Fishers Exact test, acetic and butyric acids have a weak differential association with respect to the control of *C*. *coli* strains from the different isolation sources, *P* = 0.107 and *P* = 0.097, respectively. Citric acid has no differential association with respect to the control of *C*. *coli* from the different isolation sources, *P* = 0.24.

Formic acid has differential control of *C*. *coli* strains from different isolation sources, *P* = 0.0001. Eighty percent of the strains from fecal samples required the highest formic acid concentrations (44.5 mM) for control, and 77.4% of the strains from rectal swab samples from market aged pigs required the highest formic acid concentration (44.5 mM) for control (Table 1).

Lactic acid also has differential control of *C*. *coli* strains from different isolation sources, *P* = 0.012. Thirty-three percent of the *C*. *coli* strains from retail pork chops required the highest lactic acid concentration (45.47 mM) for bacterial control (Table 1). Also, 41.7% of the *C*. *coli* strains from retail pork chops and 40% of the *C*. *coli* strains from cecal sow samples required the 2^nd^ highest concentration of lactic acid (22.74 mM) for bacterial control (Table 1). While 78.3% of the *C*. *coli* strains from cecal samples of market age pigs were controlled at 11.37 mM lactic acid (Table 1). Propionic acid showed no differential control of *C*. *coli* from different sources, *P* = 0.91, but required 27.65 mM to inhibit 73.2% of the *C*. *coli* strains from fecal and rectal swab samples (Table 1).

Table 2 presents the median, mode, range and 90^th^ percentile of the *C*. *coli* MICs and MIC_M_s for each OA.

**Table 2:** Central Tendency of the MICs and MIC_M_s^a^ for the 111 *Campylobacter coli* strains from cecal contents, feces and rectal swabs of market age pigs, cecal contents of sows and from retail pork chops against six organic acids.

Organic Acid	Median	Mode	Range	90^th^ Percentile
**Acetic Acid**				
MIC (μg/mL)	2048	2048	1024–4096	2048
MIC_M_ (mM)	34.1	34.1	17.05–68.1	34.1
**Butyric Acid**				
MIC (μg/mL)	2048	2048	1024–2048	2048
MIC_M_ (mM)	23.24	23.24	11.62–23.24	23.24
**Citric Acid**				
MIC (μg/mL)	2048	2048	1024–2048	2048
MIC_M_ (mM)	10.66	10.66	5.33–10.66	10.66
**Formic Acid**				
MIC (μg/mL)	1024	1024	512–2048	2048
MIC_M_ (mM)	22.25	22.25	11.12–44.5	44.5
**Lactic Acid**				
MIC (μg/mL)	1024	1024	512–4096	2048
MIC_M_ (mM)	11.37	11.37	5.68–45.47	22.74
**Propionic Acid**				
MIC (μg/mL)	2048	2048	256–2048	2048
MIC_M_ (mM)	27.65	27.65	3.45–27.65	27.65

^a^MIC_M_s = Molar MICs.

### Measured pH at the MICs of the *Campylobacter coli* against organic acids

**Fig 1:**
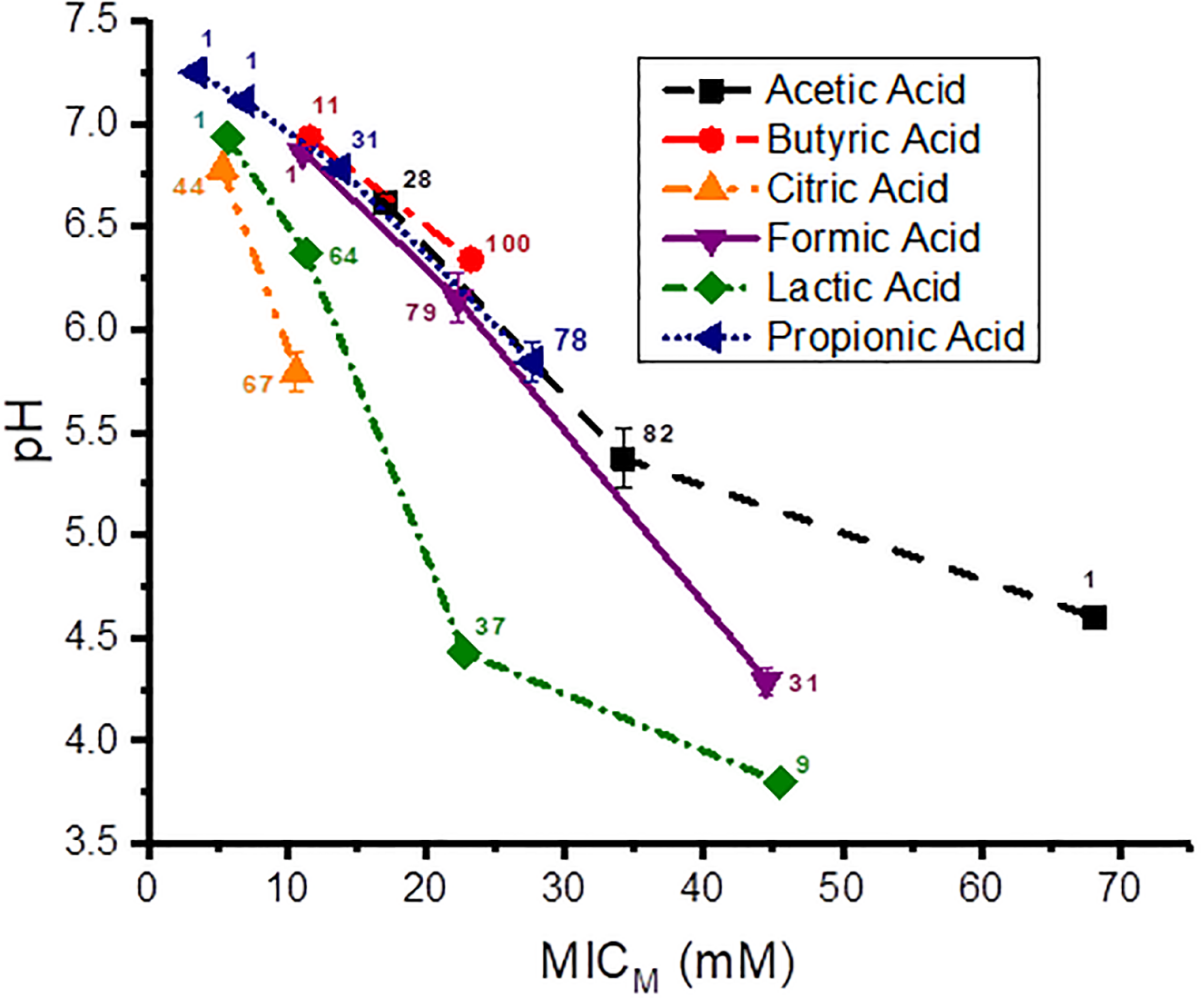
pH at the MIC_M_s of acetic, butyric, citric, formic, lactic and propionic acids for the 111 *Campylobacter coli* strains. The number of strains is shown next to each data point. Each data point is the mean and standard deviation of triplicate samples.

Since the *C*. *coli* strains behaved similarly against many of the individual different OAs, the pH determined at the *C*. *coli* MIC_M_s for all strains (n = 111) against each individual OA were combined into a single group for each OA. The pH values obtained at the *C*. *coli* MIC_M_s for the six OAs are graphically presented in Fig 1. Each data point is the mean and standard deviation of triplicate samples, and next to each data point on the graph is depicted the number of strains at each MIC_M_. The pH at the MIC_M_ for 100% of the strains against butyric, citric and propionic acids was 6.34, 5.79 and 5.84, respectively, an average pH of 5.99 ± 0.304. But the pH at the MIC_M_ for 100% of the strains against acetic, formic and lactic acids was 4.60, 4.29 and 3.80, respectively, an average pH of 4.23 ± 0.403. The pH difference for 100% of the *C*. *coli* strains against these two groups of acids is on average 1.76 pH units.

Graphical presentations showing the pH at the MIC_M_s of the *C*. *coli* strains isolated from the individual sources, cecal contents, feces and rectal swabs of market age pigs, cecal contents of sows, and from retail pork chops against the six OAs are shown for each source in S1–S5 Figs, respectively.

### Undissociated organic acid concentrations calculated at the *C*. *coli* MIC_M_s

**Fig 2:**
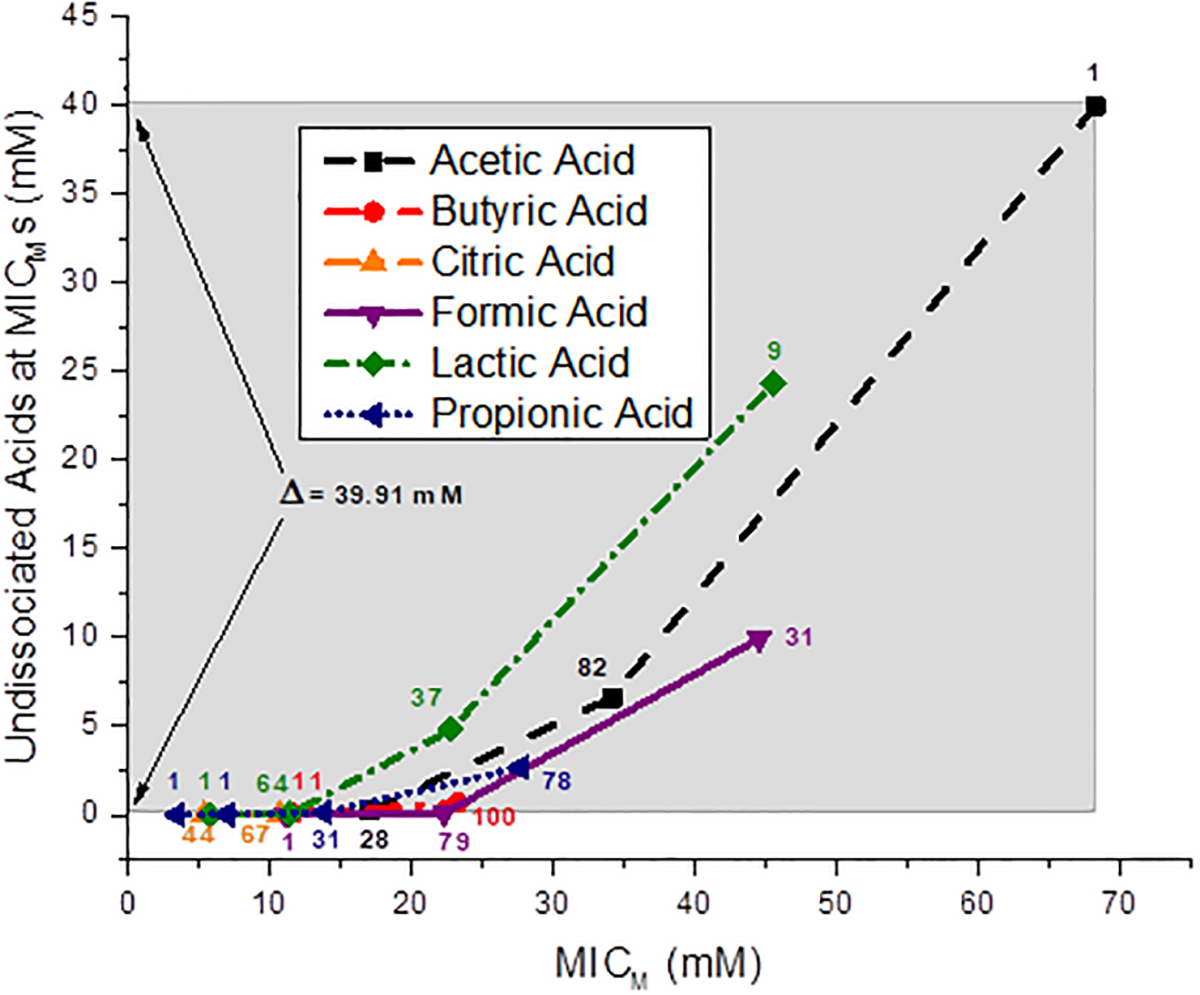
Concentration (mM) of the undissociated acids at the MIC_M_s of the 111 *Campylobacter coli* strains. The shaded band depicts the difference between the undissociated acetic and citric acid concentrations required for disinfection of 100% of the strains; Δ = 39.91 mM. The number of strains is shown next to each data point.

The results calculated by the Henderson-Hasselbalch calculation for the undissociated OA concentrations of acetic, butyric, citric, formic, lactic and propionic acids at the MIC_M_s of 111 *C*. *coli* strains are shown in Fig 2. The undissociated acetic, formic and lactic acid concentrations at the MIC_M_ for 100% of the *C*. *coli* strains tested was 39.93, 9.96 and 24.3 mM, respectively. The undissociated butyric, citric and propionic acid concentrations at the MIC_M_ for 100% of the *C*. *coli* strains tested was 0.68, 0.024 and 2.68 mM, respectively. The MIC_M_ of all 111 strains occurred at an undissociated citric acid level of 0.024 mM. The MIC_M_ of all 111 *C*. *coli* strains occurred at an undissociated acetic acid concentration of 39.93 mM. A concentration of undissociated butyric and citric acids of 0.68 and 0.024 mM was observed at 100% of the *C*. *coli* at their MIC_M_s. A difference of Δ = 39.91 mM OA levels between the MIC_M_ of 100% of the strains against acetic and citric acids is shown by the shaded band in Fig 2.

Graphical presentations showing the undissociated acid species at the MIC_M_s of the 111 *C*. *coli* strains isolated from the individual sources, cecal contents, feces and rectal swabs of market age pigs, cecal contents of sows, and from retail pork chops against the six OAs are shown for each individual source in S6–S10 Figs, respectively.

### Dissociated organic acid concentrations calculated at the *C*. *coli* MIC_M_s

**Fig 3:**
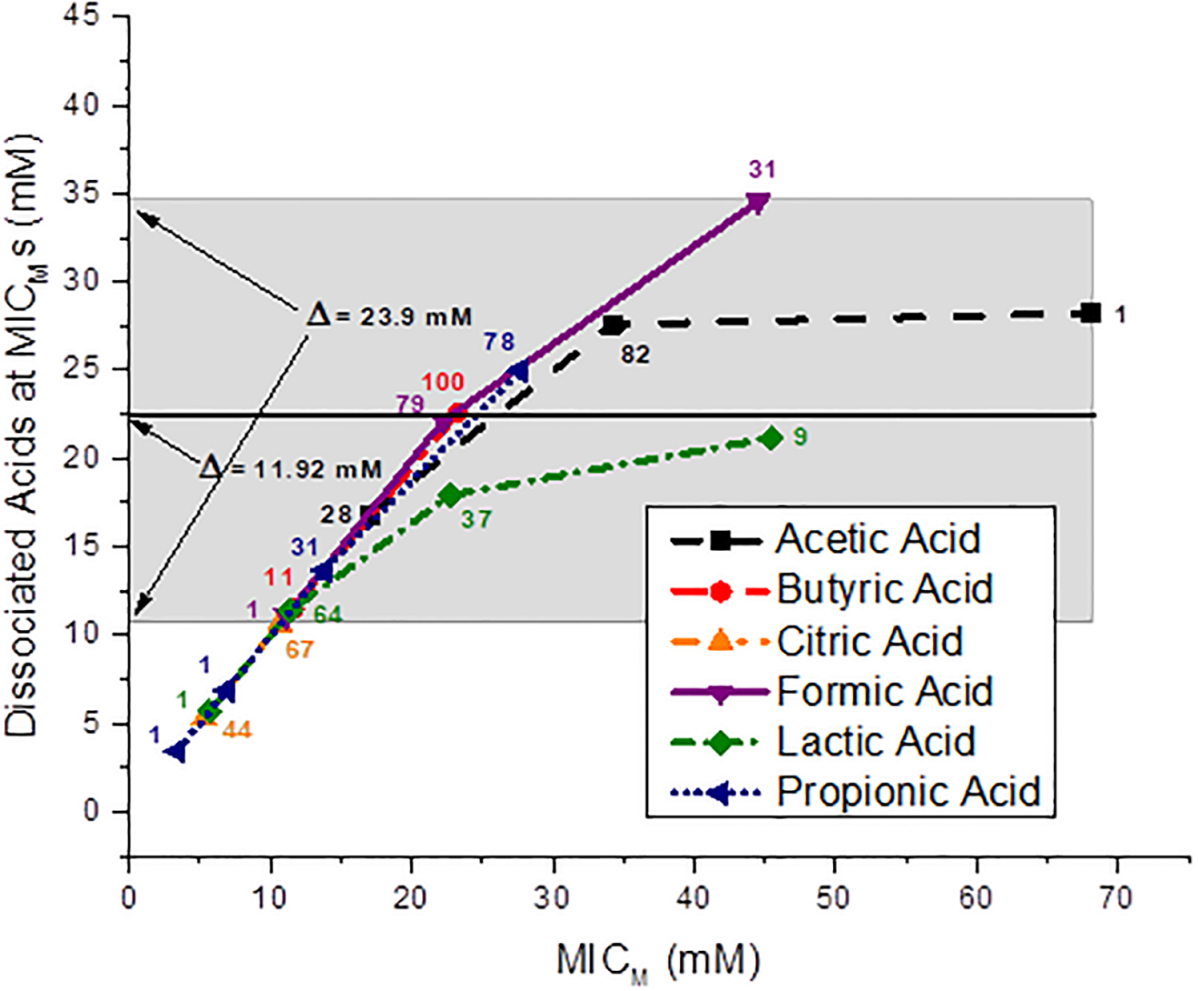
Concentration (mM) of the dissociated acids at the MIC_M_s of the 111 *Campylobacter coli* strains. The shaded band depicts the difference between the dissociated formic and citric acid concentrations required for disinfection of 100% of the strains, Δ = 23.9 mM; and the line through the 100 strain data point for butyric acid and the 67 strain data point for citric acid shows the difference in concentration for inhibition of 100% of the strains for these two acids, Δ = 11.92 mM. The number of strains is shown next to each data point.

The calculated concentrations of the dissociated OAs, acetic, butyric, citric, formic, lactic and propionic acids at the MIC_M_s of the 111 *C*. *coli* strains are shown in Fig 3. The molar dissociated OA concentrations required to produce MIC_M_s for 100% of the 111 *C*. *coli* strains by all six OAs are shown by the shaded band in Fig 3. The shaded band shows a Δ = 23.9 mM difference between the MIC_M_ of 100% of the 111 *C*. *coli* strains inhibited by citric acid and 100% of the 111 strains inhibited by the other five OAs. The MIC_M_ for 100% of the 111 strains occurs at a dissociated acid level of 10.64 mM citrate. The MIC_M_ for 100% of the 111 strains for all dissociated acids occurs at a level of 34.54 mM formate. However, only the results for the dissociated butyric and citric acids may not be affected by *C*. *coli* utilization. The concentration difference of these two dissociated acids for inhibition of 100% of the 111 *C*. *coli* results in a Δ = 11.92 mM.

Graphical presentations of the dissociated acid species at the MIC_M_s of the 111 *C*. *coli* strains isolated from the individual sources, cecal contents, feces and rectal swabs of market age pigs, cecal contents of sows, and from retail pork chops against the six OAs is shown for each individual source in S11–S15 Figs, respectively.

## Discussion

Organic acids are regularly used to decontaminate meat surfaces. But many bacterial food pathogens have the ability to adapt to varying pH environments, and decontamination strategies are often based on pH [49]. We studied six different OAs, acetic, butyric, citric, formic, lactic and propionic acids against 111 *C*. *coli* strains to evaluate the effect that pH, the undissociated and dissociated acid species had on these bacteria at their MIC_M_s.

The median MIC_M_ for acetic and propionic acids required for disinfection of the same strains are the highest and the median MIC_M_ for inhibition of the *C*. *coli* strains by butyric and formic acids have an intermediate value, while the median MIC_M_ for inhibition by citric and lactic acids have the lowest values. However, acetic, formic and lactic acids have the highest MIC_M_ values for the range of disinfection of all six OAs, and 33.3% of *C*. *coli* from retail pork chops required the highest level of lactic acid for bacterial control. While the citric acid MIC_M_s demonstrate the lowest range, and the lowest 90^th^ percentile value of 10.66 mM for inhibition of all the 111 *C*. *coli* strains. This suggests that citric acid may be the best OA for inhibiting *C*. *coli*. This is also confirmed by showing that citric acid has no differential association with respect to the control of *C*. *coli* from different isolation sources, *P* = 0.24. Conversely, citric acid has a common inhibition effect and lowest concentration required on *C*. *coli* no matter where the bacteria are isolated from.

Interestingly, it only took a pH of 6.34, 5.79 and 5.84 to inhibit 100% of these bacteria with butyric, citric and propionic acids, respectively. But with acetic, formic and lactic acids it required a pH of 4.60, 4.29 and 3.80, respectively, to inhibit the same 111 *C*. *coli* strains. This is an average of 1.76 pH unit difference between the pH required for these two groups of acids to inhibit the same 111 *C*. *coli* strains. We have reported pH differences between OAs against other Gram-negative strains, but not this large a difference. Approximately 98% of 175 *P*. *aeruginosa* strains showed a 0.98 pH unit difference when inhibited by different OAs [38]. A 0.56 pH unit difference was observed between the inhibition by different OAs for 98% of 344 *E*. *coli* O157:H7 strains [37], a 0.99 pH unit difference between different OAs was required to inhibit 100% of 138 non-O157 STEC strains [39], and a 1.1 pH unit difference was observed between four different OAs for inhibition of 95 to 100% of the same 145 *Salmonella* strains [40]. These data show that the inhibition of *C*. *coli* or the other Gram-negative bacteria are not primarily dependent on the pH of the acids, as has been suggested by others [33], but rather inhibition must be dependent on some other aspect of these acids. If indeed pH were the primary factor in bacterial inhibition, then one would expect that the MIC_M_s for the same bacteria for all the different OAs would be at the same pH value; but that is not the case. Also, we saw more acid-tolerance in *E*. *coli* O157:H7 strains [37], since they have glutamate and arginine–dependent acid-resistance systems for protection against acid stress [50].

The inhibition range for 100% of the 111 *C*. *coli* strains by all six undissociated OAs, acetic, butyric, citric, formic, lactic and propionic acids extended from 0.024 mM citric acid to 39.93 mM acetic acid, which is an undissociated acid difference of 39.91 mM across the six different OA species for the same 111 strains. Also, undissociated citric acid shows an inhibition of *C*. *coli* strains at a very dilute acid concentration of 1 μM. There appears to be no correlation as to concentration of the undissociated OAs with the MIC_M_s for the 111 *C*. *coli* strains. These results are in agreement with the four other Gram-negative foodborne pathogens we have previously studied. In 175 *P*. *aeruginosa* strains the difference between undissociated citric acid (2.53 mM) and acetic acid (21.65 mM) for inhibition of 100% of the strains at the MIC_M_s was 19.12 mM [38]. In 344 *E*. *coli* O157:H7 the difference between undissociated citric acid (2.86 mM) and acetic acid (50.63) for inhibition of 98.3% of the strains at the MIC_M_s was 47.77 mM [37]. In 138 non-O157 STECs the difference between undissociated citric acid (2.2 mM) and acetic acid (49.11 mM) for inhibition of 100% of the strains at the MIC_M_s was 46.91 mM [39], and in 145 *Salmonella* strains the difference between undissociated citric acid (2.29 mM) and acetic acid (19.0 mM) for inhibition of 100% of the strains at the MIC_M_s was 16.71 mM [40]. In all of these cases, the undissociated acid concentrations did not correlate with the MIC_M_s. Higher undissociated acid values were observed for *E*. *coli* O157:H7 and non-O157 STECs, but most likely this was a result of the glutamate and arginine–dependent acid-resistance systems inherent to those bacteria and used to protect themselves from extreme acid stress [50,51].

The inhibition of 100% of the 111 *C*. *coli* strains by the dissociated OAs was definitely a much smaller concentration range than that observed for the undissociated acids. But the inhibition concentration range shown for all six dissociated acids against *C*. *coli* is still large when compared to the dissociated OA concentration ranges against the other four Gram-negative foodborne pathogens that we previously studied. The inhibition of approximately 98% of 175 *P*. *aeruginosa* strains by dissociated citric acid (10.24 mM) and acetic acid (9.98 mM) had a concentration difference of 0.26 mM [38]. The inhibition of 98.3% of 344 *E*. *coli* O157:H7 strains by dissociated lactic acid (19.36 mM) and dissociated propionic acid (13.825 mM) had a concentration difference of 5.54 mM [37]. The inhibition of 100% of 138 non-O157 STEC strains by dissociated citric acid (19.12 mM) and lactic acid (12.93 mM) had a concentration difference of 6.19 mM [39], and the inhibition of 100% of 145 *Salmonella* strains by dissociated citric acid (19.03 mM) and propionic acid (13.67 mM) had a concentration difference of 5.36 mM [40]. The overall difference in dissociated acids required for inhibition of these four Gram-negative bacteria was from 0.26 mM to 6.19 mM. However with *P*. *aeruginosa*, we saw a large increase in the dissociated lactic acid concentration required for inhibition [38]. It is known that *P*. *aeruginosa* utilizes lactate [52,53], and the high inhibition concentration obtained for dissociated lactic acid could be expected [38]. Lactic acid is not an appropriate OA to use against *P*. *aeruginosa* [38].

Most *C*. *coli* strains from swine do not utilize citrate [54], and we see in this study the inhibition concentration for dissociated citric acid remains low, ≤ 10.64 mM. Also, *C*. *coli* were shown not to utilize butyrate [54]. This study corroborates earlier observations by demonstrating levels of dissociated butyric acid needed for inhibition of *C*. *coli* not widely different from the levels of other dissociated OAs against Gram-negative pathogens [37,39,40]. However, *C*. *coli* are known to utilize formate, lactate and propionate [55], and in a previous study approximately 13.5% of the *C*. *coli* strains utilized acetate [54]. The authors also noted the source of *C*. *coli* strains utilizing acetate was restricted to hogs [54]. Since all 111 strains are inhibited by both citric and butyric acid by ≤ 22.56 mM (knowing that *C*. *coli* does not utilize citrate or butyrate [54]), it is very interesting that 31 strains are not inhibited by dissociated formic acid until nearly 35 mM, 78 strains are not inhibited by dissociated propionic acid until about 25 mM, and 83 strains are not inhibited by dissociated acetic acid until about 28 mM. Based on our data for the dissociated acid species at the MIC_M_s of 111 *C*. *coli* strains from swine, perhaps as much as 83/111 strains (75%) of the *C*. *coli* analyzed from swine or swine products may utilize acetate.

## Conclusion

Inhibition of *Campylobacter coli* strains in this study was not primarily dependent on pH or on the concentration of undissociated OAs. The concentration of dissociated OA, butyric, citric, formic, lactic and propionic acids correlated with the MIC_M_s of 100% of the 111 *C*. *coli* strains. However, some *C*. *coli* can utilize acetate, formate, lactate and propionate, which most likely resulted in increased levels of these acids at the MICs in our studies. One may expect that a large number of bacteria could escape disinfection as a result of only a small drop in the concentration of a dissociated OA. Therefore, an OA carcass wash may not provide the expected elimination of surface bacteria if the concentration levels of the dissociated OA used is not carefully controlled. A concentration of dissociated acetic, butyric, citric, formic, lactic and propionic acids of 29, 23, 11, 35, 22 and 25 mM, respectively, should be maintained when disinfecting the *C*. *coli* strains studied here. However, due to the utilization of acetate, formate, lactate and propionate by *C*. *coli*, these four OAs would probably not be the best choice for control of *C*. *coli*. If these 4 acids are used for disinfection of *C*. *coli* bacteria the concentrations of these dissociated organic acids must be held at high enough levels to facilitate complete inhibition of the bacteria. Of the six OAs, citric acid is the most efficient at inhibiting *C*. *coli*.

## Supporting information

S1 Fig pH at the MIC_M_s of acetic, butyric, citric, formic, lactic and propionic acids for the 23 *Campylobacter coli* strains from the cecal contents of market age pigs. The number of strains is shown next to each data point. Each data point is the mean and standard deviation of triplicate samples.
(TIF)

S2 Fig pH at the MIC_M_s of acetic, butyric, citric, formic, lactic and propionic acids for the 5 *Campylobacter coli* strains from the feces of market age pigs. The number of strains is shown next to each data point. Each data point is the mean and standard deviation of triplicate samples.
(TIF)

S3 Fig pH at the MIC_M_s of acetic, butyric, citric, formic, lactic and propionic acids for the 51 *Campylobacter coli* strains from the rectal swabs of market age pigs. The number of strains is shown next to each data point. Each data point is the mean and standard deviation of triplicate samples.
(TIF)

S4 Fig pH at the MIC_M_s of acetic, butyric, citric, formic, lactic and propionic acids for the 20 *Campylobacter coli* strains from the cecal contents of sows. The number of strains is shown next to each data point. Each data point is the mean and standard deviation of triplicate samples.
(TIF)

S5 Fig pH at the MIC_M_s of acetic, butyric, citric, formic, lactic and propionic acids for the 12 *Campylobacter coli* strains from retail pork chops. The number of strains is shown next to each data point. Each data point is the mean and standard deviation of triplicate samples.
(TIF)

S6 Fig Concentration (mM) of the undissociated acids at the MIC_M_s of acetic, butyric, citric, formic, lactic and propionic acids for the 23 *Campylobacter coli* strains from the cecal contents of market age pigs. The shaded band depicts the difference between the undissociated lactic and citric acid concentrations required for disinfection of 100% of the strains; Δ = 24.3 mM. The number of strains is shown next to each data point.
(TIF)

S7 Fig Concentration (mM) of the undissociated acids at the MIC_M_s of acetic, butyric, citric, formic, lactic and propionic acids for the 5 *Campylobacter coli* strains from the feces of market age pigs. The shaded band depicts the difference between the undissociated formic and citric acid concentrations required for disinfection of 100% of the strains; Δ = 9.96 mM. The number of strains is shown next to each data point.
(TIF)

S8 Fig Concentration (mM) of the undissociated acids at the MIC_M_s of acetic, butyric, citric, formic, lactic and propionic acids for the 51 *Campylobacter coli* strains from the rectal swabs of market age pigs. The shaded band depicts the difference between the undissociated lactic and citric acid concentrations required for disinfection of 100% of the strains; Δ = 24.3 mM. The number of strains is shown next to each data point.
(TIF)

S9 Fig Concentration (mM) of the undissociated acids at the MIC_M_s of acetic, butyric, citric, formic, lactic and propionic acids for the 20 *Campylobacter coli* strains from the cecal contents of sows. The shaded band depicts the difference between the undissociated lactic and citric acid concentrations required for disinfection of 100% of the strains; Δ = 24.3 mM. The number of strains is shown next to each data point.
(TIF)

S10 Fig Concentration (mM) of the undissociated acids at the MIC_M_s of acetic, butyric, citric, formic, lactic and propionic acids for the 12 *Campylobacter coli* strains from retail pork chops. The shaded band depicts the difference between the undissociated acetic and citric acid concentrations required for disinfection of 100% of the strains; Δ = 39.86 mM. The number of strains is shown next to each data point.
(TIF)

S11 Fig Concentration (mM) of the dissociated acids at the MIC_M_s of acetic, butyric, citric, formic, lactic and propionic acids for the 23 *Campylobacter coli* strains from the cecal contents of market age pigs. The shaded band depicts the difference between the dissociated formic and citric acid concentrations required for disinfection of 100% of the strains; Δ = 16.96 mM. The number of strains is shown next to each data point.
(TIF)

S12 Fig Concentration (mM) of the dissociated acids at the MIC_M_s of acetic, butyric, citric, formic, lactic and propionic acids for the 5 *Campylobacter coli* strains from the feces of market age pigs. The shaded band depicts the difference between the dissociated formic and citric acid concentrations required for disinfection of 100% of the strains; Δ = 23.9 mM. The number of strains is shown next to each data point.
(TIF)

S13 Fig Concentration (mM) of the dissociated acids at the MIC_M_s of acetic, butyric, citric, formic, lactic and propionic acids for the 51 *Campylobacter coli* strains from the rectal swabs of market age pigs. The shaded band depicts the difference between the dissociated formic and citric acid concentrations required for disinfection of 100% of the strains; Δ = 23.9 mM. The number of strains is shown next to each data point.
(TIF)

S14 Fig Concentration (mM) of the dissociated acids at the MIC_M_s of acetic, butyric, citric, formic, lactic and propionic acids for the 20 *Campylobacter coli* strains from the cecal contents of sows. The shaded band depicts the difference between the dissociated formic and citric acid concentrations required for disinfection of 100% of the strains; Δ = 23.9 mM. The number of strains is shown next to each data point.
(TIF)

S15 Fig Concentration (mM) of the dissociated acids at the MIC_M_s of acetic, butyric, citric, formic, lactic and propionic acids for the 12 *Campylobacter coli* strains from retail pork chops. The shaded band depicts the difference between the dissociated acetic and citric acid concentrations required for disinfection of 100% of the strains; Δ = 17.59 mM. The number of strains is shown next to each data point.
(TIF)
