# Supplementary figures and images for: Interactions of organic acids with Campylobacter coli from swine
Source: PLoS One. 2018 Aug 10;13(8):e0202100. doi: 10.1371/journal.pone.0202100 (PMC6086449; doi:10.1371/journal.pone.0202100)

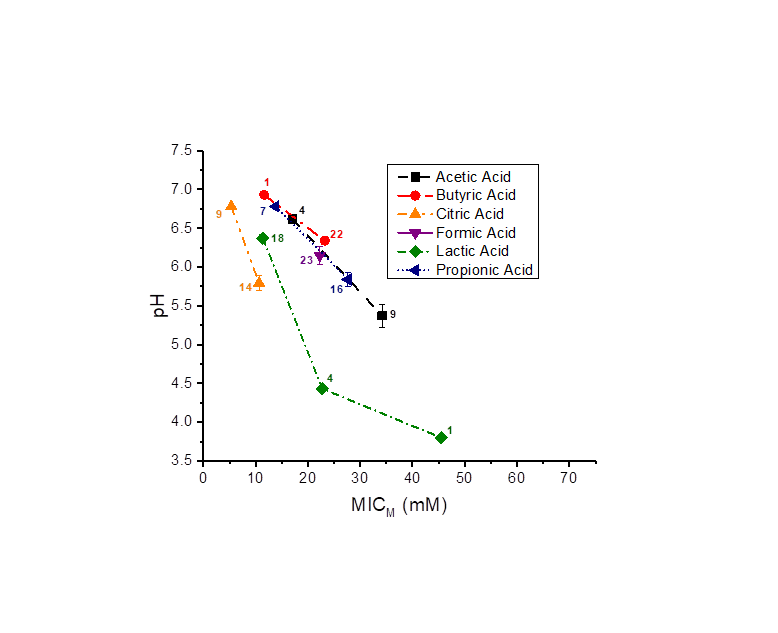

Supplement: S1 Fig — The number of strains is shown next to each data point. Each data point is the mean and standard deviation of triplicate samples. (TIF) [file pone.0202100.s001.tif]

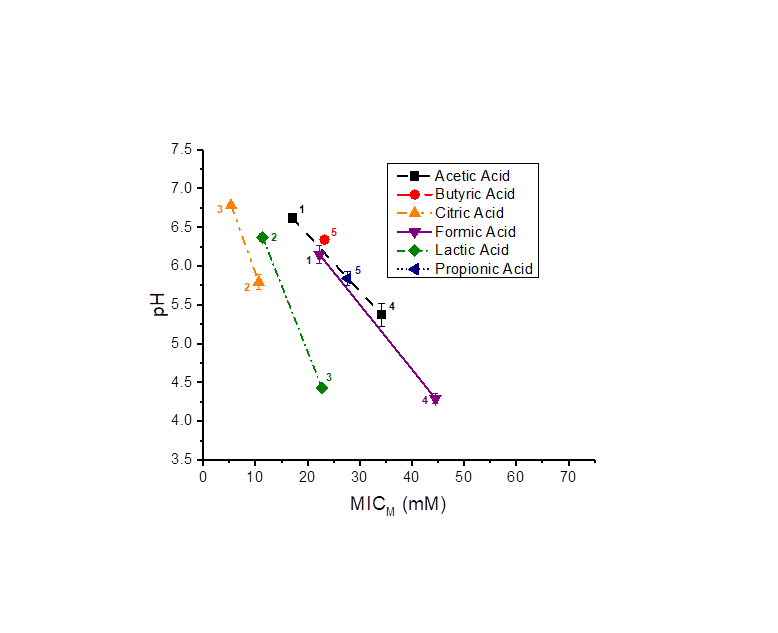

Supplement: S2 Fig — The number of strains is shown next to each data point. Each data point is the mean and standard deviation of triplicate samples. (TIF) [file pone.0202100.s002.tif]

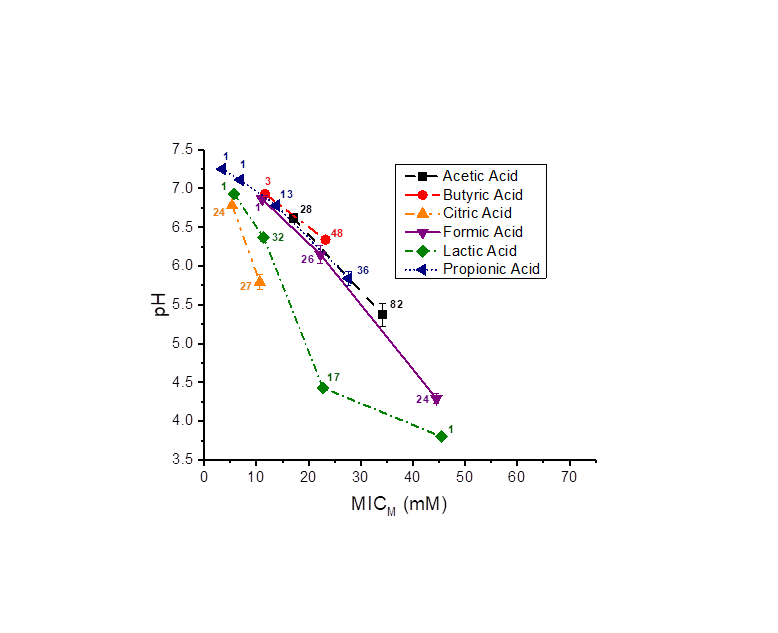

Supplement: S3 Fig — The number of strains is shown next to each data point. Each data point is the mean and standard deviation of triplicate samples. (TIF) [file pone.0202100.s003.tif]

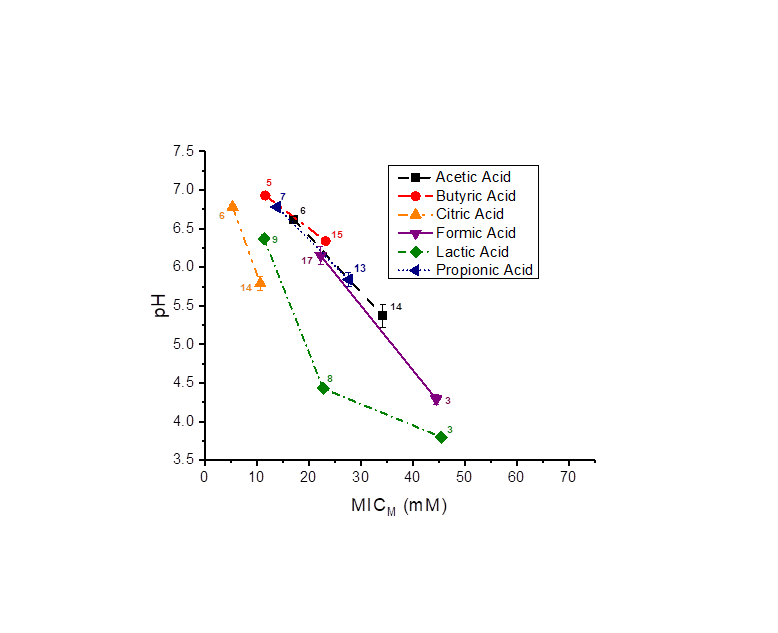

Supplement: S4 Fig — The number of strains is shown next to each data point. Each data point is the mean and standard deviation of triplicate samples. (TIF) [file pone.0202100.s004.tif]

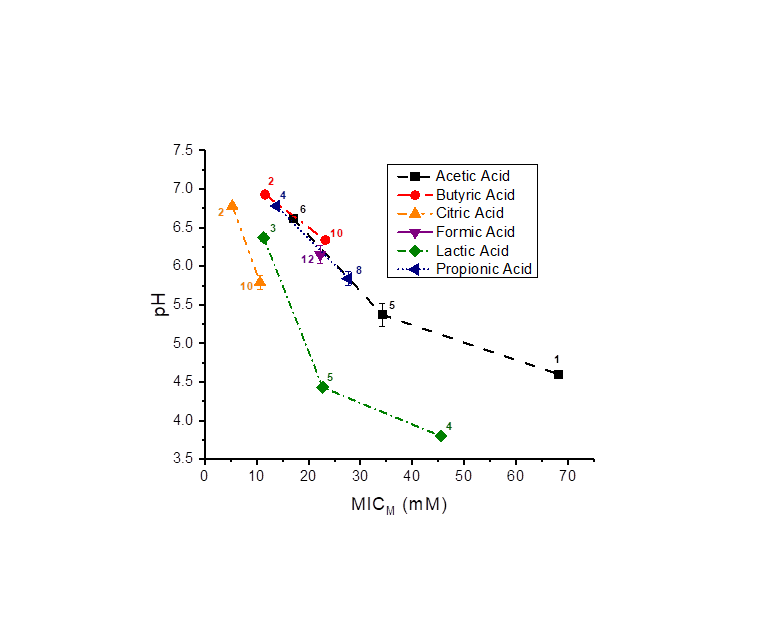

Supplement: S5 Fig — The number of strains is shown next to each data point. Each data point is the mean and standard deviation of triplicate samples. (TIF) [file pone.0202100.s005.tif]

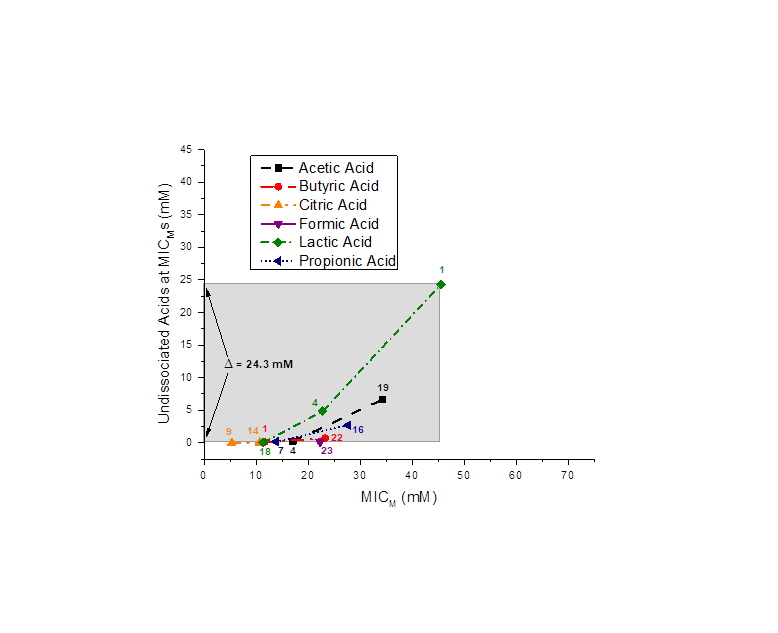

Supplement: S6 Fig — The shaded band depicts the difference between the undissociated lactic and citric acid concentrations required for disinfection of 100% of the strains; Δ = 24.3 mM. The number of strains is shown next to each data point. (TIF) [file pone.0202100.s006.tif]

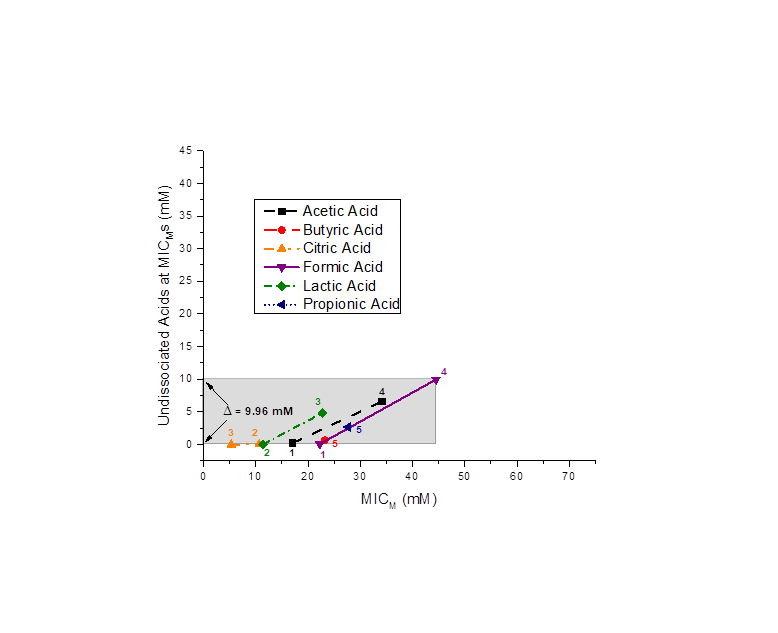

Supplement: S7 Fig — The shaded band depicts the difference between the undissociated formic and citric acid concentrations required for disinfection of 100% of the strains; Δ = 9.96 mM. The number of strains is shown next to each data point. (TIF) [file pone.0202100.s007.tif]

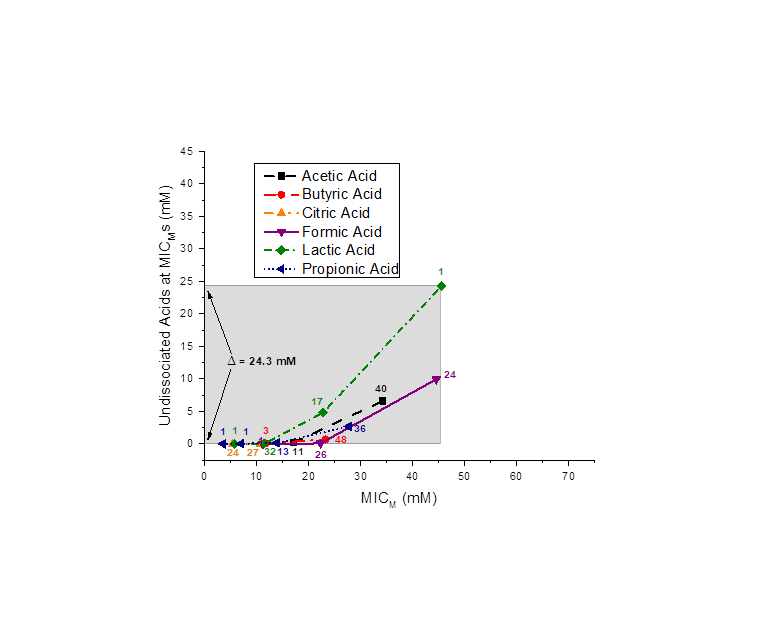

Supplement: S8 Fig — The shaded band depicts the difference between the undissociated lactic and citric acid concentrations required for disinfection of 100% of the strains; Δ = 24.3 mM. The number of strains is shown next to each data point. (TIF) [file pone.0202100.s008.tif]

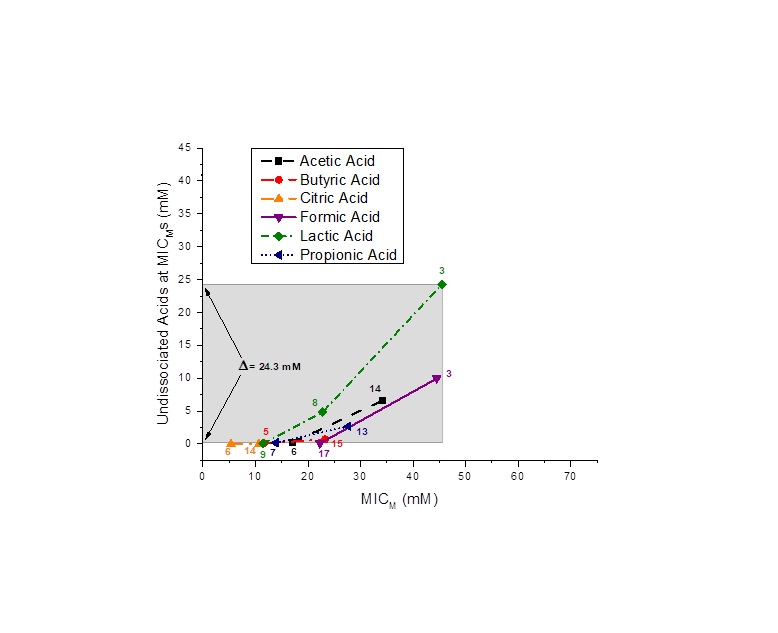

Supplement: S9 Fig — The shaded band depicts the difference between the undissociated lactic and citric acid concentrations required for disinfection of 100% of the strains; Δ = 24.3 mM. The number of strains is shown next to each data point. (TIF) [file pone.0202100.s009.tif]

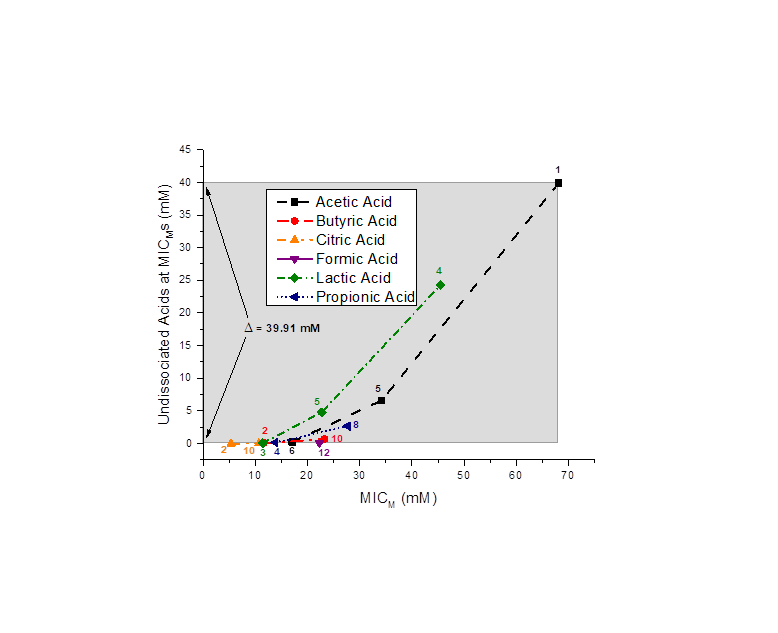

Supplement: S10 Fig — The shaded band depicts the difference between the undissociated acetic and citric acid concentrations required for disinfection of 100% of the strains; Δ = 39.86 mM. The number of strains is shown next to each data point. (TIF) [file pone.0202100.s010.tif]

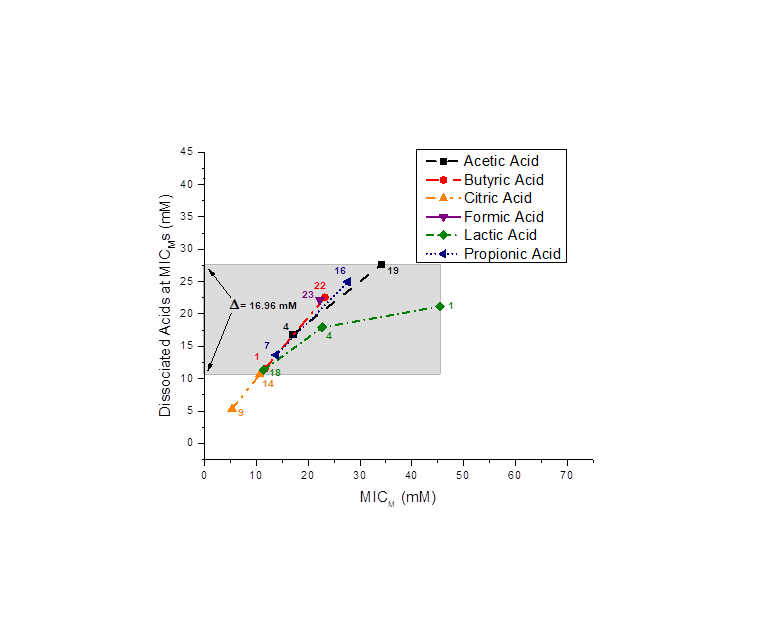

Supplement: S11 Fig — The shaded band depicts the difference between the dissociated formic and citric acid concentrations required for disinfection of 100% of the strains; Δ = 16.96 mM. The number of strains is shown next to each data point. (TIF) [file pone.0202100.s011.tif]

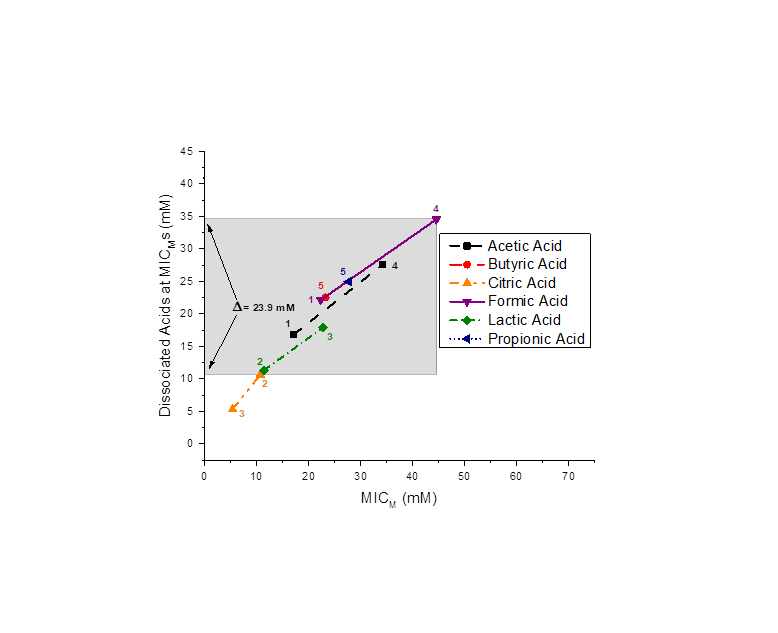

Supplement: S12 Fig — The shaded band depicts the difference between the dissociated formic and citric acid concentrations required for disinfection of 100% of the strains; Δ = 23.9 mM. The number of strains is shown next to each data point. (TIF) [file pone.0202100.s012.tif]

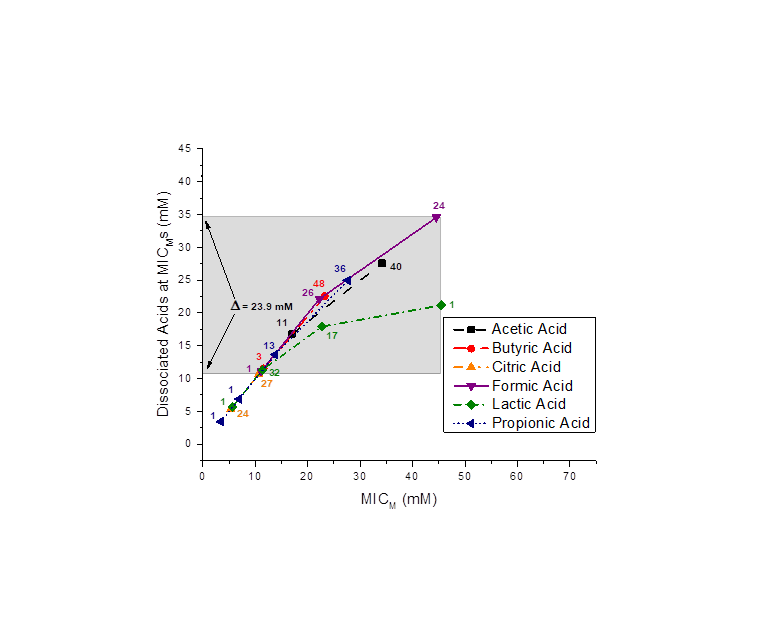

Supplement: S13 Fig — The shaded band depicts the difference between the dissociated formic and citric acid concentrations required for disinfection of 100% of the strains; Δ = 23.9 mM. The number of strains is shown next to each data point. (TIF) [file pone.0202100.s013.tif]

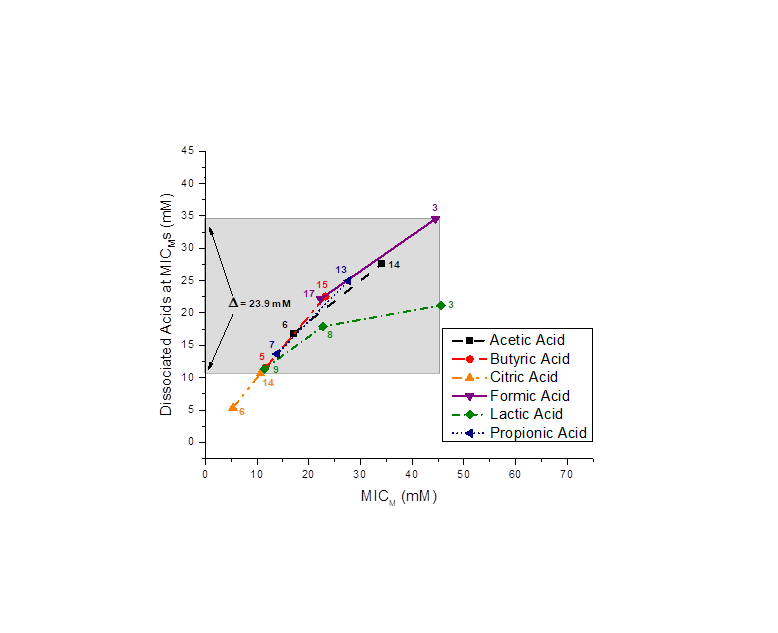

Supplement: S14 Fig — The shaded band depicts the difference between the dissociated formic and citric acid concentrations required for disinfection of 100% of the strains; Δ = 23.9 mM. The number of strains is shown next to each data point. (TIF) [file pone.0202100.s014.tif]

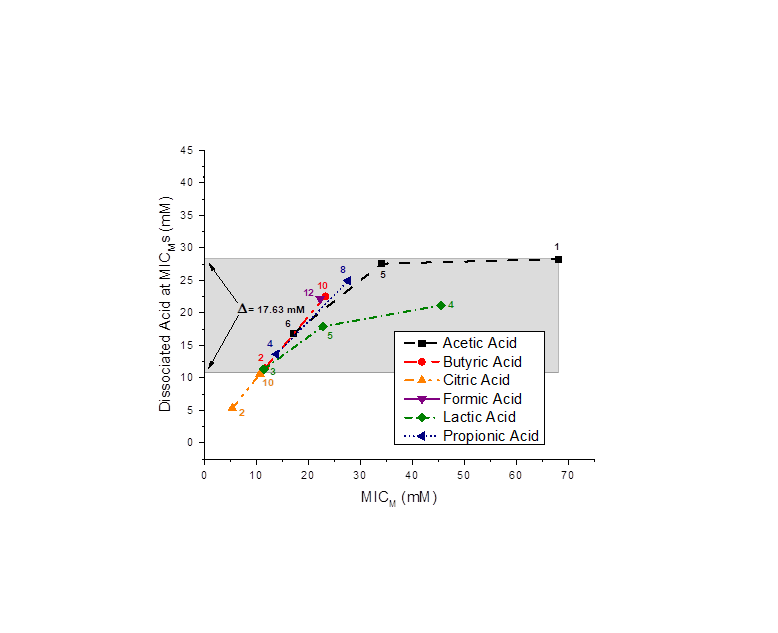

Supplement: S15 Fig — The shaded band depicts the difference between the dissociated acetic and citric acid concentrations required for disinfection of 100% of the strains; Δ = 17.59 mM. The number of strains is shown next to each data point. (TIF) [file pone.0202100.s015.tif]
